# Economic costs and health-related quality of life for hand, foot and mouth disease (HFMD) patients in China

**DOI:** 10.1371/journal.pone.0184266

**Published:** 2017-09-21

**Authors:** Yaming Zheng, Mark Jit, Joseph T. Wu, Juan Yang, Kathy Leung, Qiaohong Liao, Hongjie Yu

**Affiliations:** 1 Key Laboratory of Surveillance and Early-warning on Infectious Disease, Division of Infectious Disease, Chinese Center for Disease Control and Prevention, Beijing, China; 2 Modelling and Economics Unit, Public Health England, London, United Kingdom; 3 Department of Infectious Disease Epidemiology, London School of Hygiene & Tropical Medicine, London, United Kingdom; 4 WHO Collaborating Centre for Infectious Disease Epidemiology and Control, School of Public Health, Li Ka Shing Faculty of Medicine, the University of Hong Kong, Hong Kong Special Administrative Region, China; 5 School of Public Health, Fudan University, Key Laboratory of Public Health Safety, Ministry of Education, Shanghai, China; Boston University School of Public Health, UNITED STATES

## Abstract

**Background:**

Hand, foot and mouth disease (HFMD) is a common illness in China that mainly affects infants and children. The objective of this study is to assess the economic cost and health-related quality of life associated with HFMD in China.

**Method:**

A telephone survey of caregivers were conducted in 31 provinces across China. Caregivers of laboratory-confirmed HFMD patients who were registered in the national HFMD enhanced surveillance database during 2012–2013 were invited to participate in the survey. Total costs included direct medical costs (outpatient care, inpatient care and self-medication), direct non-medical costs (transportation, nutrition, accommodation and nursery), and indirect costs for lost income associated with caregiving. Health utility weights elicited using EuroQol EQ-5D-3L and EQ-Visual Analogue Scale (VAS) were used to calculate associated loss in quality adjusted life years (QALYs).

**Results:**

The subjects comprised 1136 mild outpatients, 1124 mild inpatients, 1170 severe cases and 61 fatal cases. The mean total costs for mild outpatients, mild inpatients, severe cases and fatal cases were $201 (95%CI $187, $215), $1072 (95%CI $999, $1144), $3051 (95%CI $2905, $3197) and $2819 (95%CI $2068, $3571) respectively. The mean QALY losses per HFMD episode for mild outpatients, mild inpatients and severe cases were 3.6 (95%CI 3.4, 3,9), 6.9 (95%CI 6.4, 7.4) and 13.7 (95%CI 12.9, 14.5) per 1000 persons. Cases who were diagnosed with EV-A71 infection and had longer duration of illness were associated with higher total cost and QALY loss.

**Conclusion:**

HFMD poses a high economic and health burden in China. Our results provide economic and health utility data for cost-effectiveness analysis for HFMD vaccination in China.

## 1. Introduction

Hand, foot and mouth disease (HFMD) is an acute communicable disease caused by enteroviruses, most commonly enterovirus A71 (EV-A71) and coxsackie virus A16 (CV-A16). [1] Most HFMD cases are children under five years of age, with those younger than three years of age being the most susceptible.[2–3] HFMD has been the leading cause of morbidity among all nationally notifiable diseases since 2009 and become a major public health concern which is associated with a substantial burden of disease in China. [4–5]

EV-A71 vaccines are the first vaccines developed against HFMD and became commercially available in China in 2016 [6]. Although EV-A71 vaccination may be the most effective way for alleviating the disease burden of HFMD, their inclusion into the national immunization program depends on their cost-effectiveness. A key input for cost-effectiveness analysis is the economic costs and the effect on health-related quality of life (HRQoL) associated with HFMD. However, most of the relevant cost data in the current literature are only available for high-income provinces such as Shanghai and Jiangsu [7, 8]. Few studies have been conducted in less developed provinces with higher HFMD incidence, such as Hunan and Guangxi. [9] Regarding HRQoL of HFMD patients, there has only been one relevant study in a rural village in Jiangsu. [8] The cost and HRQoL of HFMD patients in developed provinces are unlikely to be representative of those in less developed regions. To address this knowledge gap, we conducted a national telephone survey among caregivers of laboratory-confirmed HFMD cases who were registered in the national HFMD enhanced surveillance database to estimate the direct costs, indirect costs and quality-adjusted life years (QALY) lost associated with HFMD.

## 2. Methods

### 2.1 National HFMD enhanced surveillance database

The national HFMD enhanced surveillance database was established in 2008 to monitor the spatial, temporal and population distribution of HFMD cases as well as serotype trends and clinical severity of HFMD across mainland China. All medical institutions are required to report clinically confirmed HFMD cases, including both mild and severe cases. Mild cases are defined as patients with mouth vesicles and skin rashes on the hands and feet, fever, but no complications; severe cases are defined as patients with neurological, respiratory, or circulation complications besides the clinical manifestations of mild cases [10]. In each county/district, specimens (stool, rectal or throat swabs) of the first five clinically confirmed mild HFMD cases in each week and of all severe and fatal cases are collected and sent to local Centers for Disease Control and Prevention (CDCs) for pathogen testing using real-time fluorescence relative quantitative reverse transcription polymerase chain reaction (rRT-PCR). Demographics (e.g., age, gender), clinical details (e.g., date of symptoms onset, date of diagnosis, severity, and date of death), and contact information (e.g., telephone number) of each clinically confirmed case are recorded in the national HFMD enhanced surveillance database. For cases that are laboratory confirmed to be positive for enteroviruses, their serotyping results (i.e., EV-A71, CV-A16 or other enteroviruses (OEVs)) are also available.

### 2.2 Participants

HFMD cases reported in the national HFMD enhanced surveillance database during 2012–2013 were eligible for our study if they met the following inclusion criteria: (i) aged six months to five years; (ii) laboratory confirmed HFMD cases; (iii) without other underlying diseases. Previous studies suggest that the costs of HFMD varied significantly by level of severity. [7, 11–12] Consequently, participants in our study were stratified into four groups of severity: mild cases who were outpatients (mild outpatients), mild cases who were hospitalized (mild inpatients), severe cases and fatal cases.

A total of 192,859 laboratory-confirmed HFMD cases were reported in the National surveillance system in 2012–2013. Among those, 173,911 were aged between six months and five years. Due to budget constraint, we aimed to enroll approximately 3,500 HFMD cases, which corresponded to around 2.0% of all laboratory-confirmed HFMD cases in 2012–2013. We reached our target sample size after contacting 29,810 laboratory-confirmed cases. Among these 3,500 subjects, 1136, 1124 and 1170 were mild outpatients, mild inpatients and severe cases, respectively. The expected standard error was 23.2 Chinese Yuan (CNY), 71.1 CNY and 125.3 CNY for economic costs of mild outpatients, mild inpatients and severe cases. [11] Since the total number of fatal cases was limited, all eligible cases were included in our survey. Our sampling scheme aimed to recruit equal number of participants from each of the seven regions of China. If there were insufficient candidates in a given region, then we recruited from its neighboring regions in order to reach our target sample sizes. The distribution of age, gender and aetiology (EV-A71, CV-A16 and OEV) of participants recruited in each severity stratum (except fatal cases) matched with that reported in the national HFMD enhanced surveillance database in 2013.

### 2.3 Telephone survey

Telephone surveys were conducted between 1 Dec 2013 and 15 Jan 2014 among caregivers of HMFD patients. Each telephone number was dialed up to five times on different days before being classified as unreachable. After explaining the study and obtaining verbal consent, we verified both the demographic and clinical details of each patient. Then the interviewees were encouraged to recall information about costs and HRQoL related to that HFMD episode. The questionnaire contained questions about: 1) disease information (e.g., complications, duration of illness); 2) treatment (e.g., number of outpatient visits, duration of hospitalization); 3) costs associated with the first four outpatient visits and first two hospitalizations (e.g., costs of prescribed medications and examinations, transportation, food, accommodation, nutrition and snacks, nursery, number of caregivers and days-off-work) and self-medication (S1 Table). Interviewees were asked to recall total medical costs, which were specified on their medical bills (including both out-of-pocket expenses and payments by insurers). If the interviewees could not recall the costs, they were asked to select from a list of cost ranges (S1 Table). The lower limit of the selected range was used in the base-case analyses, while the upper limit of the range was used in the sensitivity analyses.

Alongside the aforementioned economic cost survey, EuroQoL EQ-5D-3L (hereafter “EQ-5D”) instruments were used to solicit the HRQoL of patients during their HFMD episode. HRQoL is defined as those aspects of self-perceived well-being that are related to or affected by the presence of disease or treatment. [13] As an important parameter in cost-effectiveness analysis, HRQol for HFMD patients measures the effect of disease on health states.

The EQ-5D is a widely used generic instrument for measuring non-disease-specific HRQoL, comprising a descriptive system (with five dimensions: mobility, self-care, usual activity, pain/discomfort, and anxiety/depression) and a visual analogue scale (VAS) (i.e., a scale from 0 to 100, with the extreme values representing death and full health, respectively). Subjects in this study were children aged between six months and five years. As such, the slightly altered proxy version (proxy 2, in which proxies (parents or caregivers) are asked to rate how they think their children would rate their HRQoL) (S1 Table) in simplified Chinese was used in this survey. The mobility dimension was not scored for patients younger than 18 months because not all children at these ages were able to move independently. The self-care dimension was not scored for all patients because most children under the age of five were unlikely to care for themselves. For those participants, the mobility and self-care dimensions both were given the value of “no problems” in the baseline analysis and “severe problems” in the sensitivity analysis.

To assess the reliability of caregiver-reported costs compared to costs from medical accounts, we conducted a review of medical accounts of 15 severe HMFD patients reported in Xi’an Children hospital, a designated tertiary hospital for HFMD treatment in Northwest China.

### 2.4 Data analysis

The economic cost of an HFMD episode was calculated as the sum of direct medical costs (prescription and over-the-counter medicines, outpatient and inpatient treatment), direct non-medical costs (transportation, accommodation, food, nutrition, snacks and nursery care) and indirect costs (productivity loss of caregivers due to lost workdays). Indirect costs were calculated using the human capital method, by multiplying mean daily income per capita with the days off work taken by caregiving parents. [14] Indirect costs due to premature deaths were not considered.

Responses to the EQ-5D descriptive system were converted into health utilities using a China-specific value set. Several such sets have been developed; we used the set estimated using ordinary least squares regression fitted to time trade-offs for respondents presented with different health states (the “N3 model” in [15]). QALY loss was then estimated by multiplying the difference in health utility between the HFMD episode and full health (assuming the health utility equals to one) with the reported duration of HFMD illness (S1 File). We expressed the results as QALY loss per 1000 cases (QALY loss multiple 1000) instead of QALY loss per case because the magnitude of the latter was small.

The crude mean cost and HRQoL estimates among our subjects were not representative because the geographical distribution of HFMD cases in the national database was different from that of our subjects. As such, we weighted the region-specific cost and QALY loss estimates by the number of cases in each geographic region as reported in the national HFMD enhanced surveillance database (S2 Table). Detailed equations are shown in S1 File.

Statistical analyses were performed using STATA 12.0 (StataCorp, Texas, USA). Costs in 2012 were inflated to 2013 values using the medical consumer price index for China (1.03) [16] and presented in US dollar using an exchange rate at 6.2 [17]. Bootstrap multiple linear regression (1,000 replications) was used to analyze the determinants of the total cost. Although the distribution of QALY loss was left-skewed, the sample size was large. Thus, we used t-test and ANOVA to analyze the differences in QALY losses between groups. The significance level was set at 0.05.

### 2.5 Ethical approval and informed consent

During the interview, the interviewers explained the objective and other details of the study before starting the interview. Once the interviewees agreed to participant the interview, the interviewers started the survey. All of interviews were conducted by telephone, hence we used the verbal consents instead the written ones. All verbal informed consents and the telephone survey dialogues were documented in CDs. All the study protocol were submitted to and reviewed by the institutional review board of China CDC and the ethical approval (Approval number: 201417) was acquired.

## 3. Results

### 3.1 Sample selection

**Fig 1:**
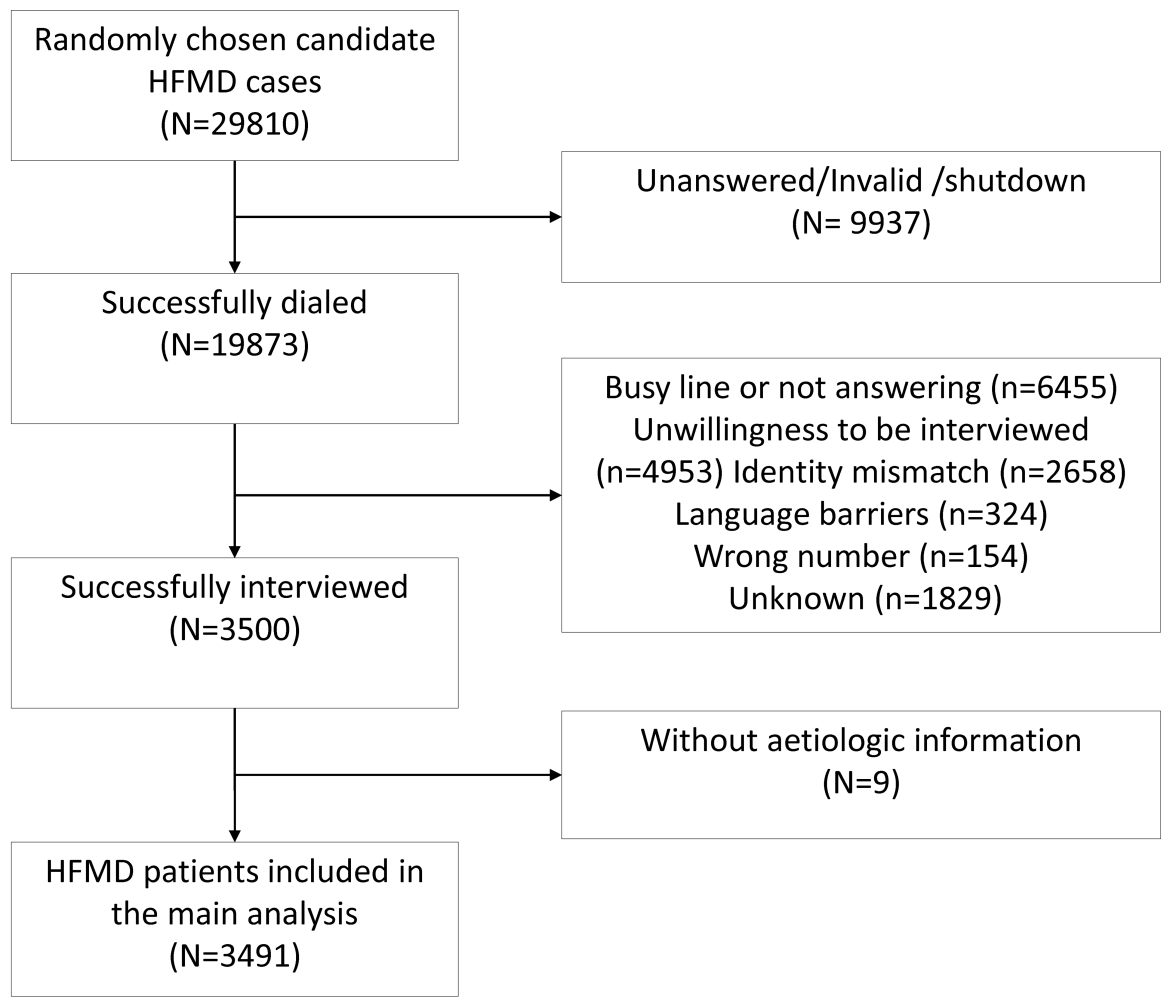
Flowchart of sample selection trough telephone interview.

We contacted 29,810 eligible HFMD cases by phone. Among these 29,810 candidates, 9937 cases were unreachable because their phone number were invalid, their lines were shutdown, or our calls were unanswered. We interviewed 3,500 cases after establishing contact with 19,873 candidates, i.e. a response rate of 17.6% (3,500/19,873). Reasons for non-participation (n = 16373) included busy line or not answering (n = 6455), unwillingness to be interviewed (n = 4953), identity mismatch (n = 2658), language barriers (n = 324), wrong number (n = 154), and unknown (n = 1829). Nine fatal cases were excluded because the national database did not have their aetiologic details. The subjects for our main analysis comprised 3,491 laboratory confirmed HFMD cases. (Fig 1).

### 3.2 Demographic and clinical information

The demographic and clinical information of HFMD patients in the telephone survey are show in Table 1. Most of the 3491 HFMD cases were male (66%), under three years of age (68%), and diagnosed with EV-A71 (51%). The duration of illness for severe HFMD patients (12.0 days, SD 7.1 days) were longer than that of mild outpatients (7.1 days, SD 4.0 days), mild inpatients (8.8 days, SD 4.5 days), and fatal cases (7.8 days, SD 5.6 days). (S1 Fig)

**Table 1:** Demographic and clinical information of study participants (N = 3491) (n, %).

	Mild outpatient N = 1136	Mild inpatient N = 1124	Severe N = 1170	Fatal N = 61	Total
Gender					
Male	737(65)	743(66)	777(66)	35(57)	2292(66)
Age group					
≤3 years	727(63)	767(68)	873(75)	44(72)	2411(69)
>3 years	409(36)	357(32)	297(25)	17(28)	1080(31)
Geographic regions					
Northeast	171(15)	180(16)	41(3)	13(21)	405(12)
Northwest	164(14)	140(13)	197(17)	3(5)	504 (14)
North	164(14)	241(21)	100(9)	2(3)	507 (15)
Central	162(14)	136(12)	225(19)	5(8)	528 (15)
Southwest	144(13)	130(12)	221(19)	13(21)	508 (15)
East	173(15)	153(14)	169(14)	10(16)	505 (14)
South	158(14)	144(13)	217(19)	15(25)	534 (15)
Aetiologic classification					
EV-A71	451(40)	484(43)	798(68)	54(88)	1787(51)
CV-A16	191(17)	172(15)	46(4)	1(2)	410(12)
OEV	494(43)	468(42)	326(28)	6(10)	1294 (37)
Duration of illness/days					
≤5	237(21)	55(5)	26(2)	24(39)	342(10)
6–10	688(61)	710(63)	431(37)	17(28)	1846(53)
11–15	138(12)	225(20)	384(33)	10(16)	757(22)
16–20	52(5)	99(9)	204(17)	6(10)	361(10)
≥21	21(2)	35(3)	125(11)	4(7)	185(5)

EV-A71: enterovirus A 71, CV-A16: coxsackievirus A 16. OEV: other enterovirus.

### 3.3 Self-reported economic cost

**Fig 2:**
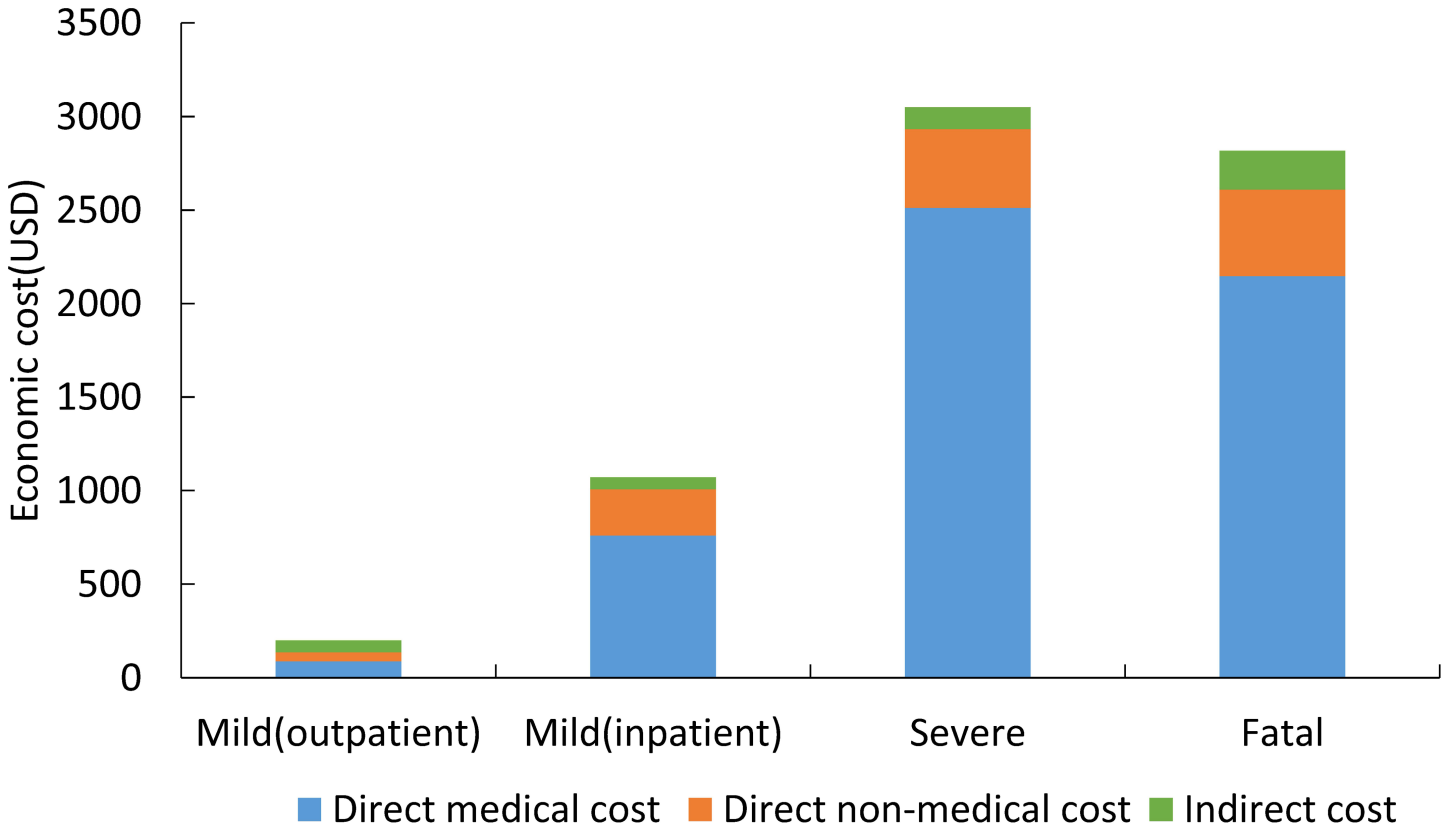
Self-reported economic costs per episode for HFMD patients in China.

Recalled self-reported costs associated with HFMD illness are shown in Fig 2. The total costs for mild outpatients, mild inpatients, severe cases and fatal cases (after weighting by geographic distribution of cases) were $196 (95%CI $75, $318), $990 (95%CI $431, $1549), $3084 (95%CI $813, $5354) and $2348 (95%CI $1006, $3689) respectively. Direct medical costs accounted for 44%, 71%, 82% and 76% of the total costs for mild outpatient, mild inpatients, severe cases and fatal cases. Multiple linear regression showed that costs were higher in patients who had longer duration of illness, were diagnosed with EV-A71 infection, and were three years old or younger. (S3 Table)

The cost estimates from our medical accounts review survey were 22.7% (95% CI 9.9% to 35.6%) higher than that in our telephone survey (see S4 Table for details).

### 3.4 Health-related quality of life

Table 2 summarizes the results of the HRQoL survey. Weighted QALY losses for mild outpatients, mild inpatients and severe cases using the EQ-5D-3L were 3.5 (95%CI 3.4, 3.9), 6.9 (95%CI 6.4, 7.4) and 13.7 (95%CI 12.9, 14.5) per 1000 cases respectively. Mild inpatients and outpatients under the age of three had higher QALY loss compared to their older counterparts even if “no problem” was assigned to the “mobility” dimension for those younger than 18 months. (*P*<0.01) Mild inpatients and severe patients with EV-A71 infection had higher QALY loss. (*P*<0.001) (S5 Table, S2 and S3 Figs). Among our subjects, HFMD had relatively greater impact on their levels of anxiety/depression and pain/discomfort compared to their mobility and usual activity.

**Table 2:** Health related quality of life for HFMD patients in China.

	Mild outpatient N = 1136		Mild inpatient N = 1124		Severe N = 1170	
	Mean(95%CI)	Median(IQR)	Mean(95%CI)	Median(IQR)	Mean(95%CI)	Median(IQR)
Health utility elicited by EQ-5D	0.83(0.82,0.83)	0.78(0.78,1.00)	0.74(0.73,0.75)	0.78(0.64,0.87)	0.61(0.59,0.62)	0.62(0.44,0.78)
Health utility elicited by EQ-VAS	75(74,76)	80(60,90)	68(67,69)	70(50,80)	56(54,57)	60(0,100)
QALY loss per 1000 cases	3.6(3.4, 3.9)	2.7(0.2, 3.9)	6.9(6.4, 7.4)	4.8(2.5, 8.5)	13.7(12.9, 14.5)	9.6(0,77.3)
Weighted QALY loss per 1000 cases	3.5(1.7,5.2)	3.3(1.9,4.7)	6.4(3.6, 9.3)	6.2(3.0, 10.2)	13.7(3.9, 23.5)	9.3(4.3, 24.8)
% of problem reported in each dimension						
Mobility^#^	7		18		44	
Usual activity	18		33		54	
Pain/discomfort	61		74		79	
Anxiety/depression	62		74		87	

^#^ “No problem” was given to “self-care” for all participants and to “mobility” for those who were younger than 18 months.

### 3.5 Sensitivity analysis

Among our subjects, caregivers of 33%, 49%, 48% and 30% of mild outpatients, mild inpatients, severe cases and fatal cases were unable to recall the exact costs for at least one item. As such, they were asked to choose from a list of cost ranges that we provided. When the upper-limits of the cost ranges were used in our analyses, the total costs for mild outpatients, mild inpatients, severe cases and fatal cases increased by 96%, 17%, 9% and 10%, respectively.

Eighteen percent, 21% and 25% of mild outpatients, mild inpatients and severe patients were younger than 18 months. In our baseline analysis, we assigned “no problem” to the mobility dimension of these cases. QALY loss estimates from EQ-5D for mild outpatients, mild inpatients and severe cases increased by 153% (9.1 vs. 3.6), 100% (13.8 vs. 6.9) and 69% (23.1 vs. 13.7) per 1000 cases, respectively, if (i) “severe problem” was assigned to the mobility dimension of these cases and (ii) “severe problem” was assigned to the self-care dimension of all subjects.

## 4. Discussion

This is the first national study in China for estimating the economic costs and HRQoL of HFMD patients by conducting telephone surveys of more than 3,000 caregivers of patients across the country. We found that the economic cost per HFMD case ranges from around $200 for a mild outpatient case to over $3,000 for a severe case, with most costs being direct medical costs. The economic burden of mild outpatients, mild inpatients, severe cases and fatal cases corresponded to 2.8%, 15.2%, 43.1% and 39.8% of the GDP per capita of China in 2013 [18], respectively.

Several studies have examined the economic costs associated with HFMD in China, but they were restricted to relatively developed provinces in east China such as Shanghai [7, 19], Jiangsu [8, 20], Zhejiang [21, 22] and Shandong [23–24]. Our study is the first to assess the economic cost and HRQoL of HFMD patients from all seven geographic regions across China, including relatively underdeveloped provinces with higher incidence of HFMD (such as Hunan and Guangxi). [9] It also gave the first national representative results of economic and QALY loss on HFMD, which provided a nationwide picture of the economic burden and QALY loss for HFMD cases with different age, gender, severity, aetiology, geographies and duration of illness.

Our cost estimates are comparable to those in previous surveys (when inflated to 2013 figures). The costs of mild outpatients in our study were similar to those from the studies in Changchun [25] ($252 vs. $260 for the Northeast region in our study) and Jinan [24] ($270 vs. $251 for the East region in our study). For mild inpatients, our results were similar to those reported in the Changchun [25] ($1023 vs $922) and Shanghai [19] ($1051 vs $933 for the East region in our study). For severe patients, the costs in this study were similar to those in the Wuxi study [20] ($3546 vs $3,059 for the East region in our study).

In this study, we obtained the self-reported recall costs from telephone interview. Unlike our study, most economic studies of illness conducted in developed countries used national medical insurance database. However, a nationwide medical insurance dataset was not available in China. As such, self-reported costs from patients is the only practical source of nationally representative data in China. Given that self-reported costs are subject to recall bias, we conducted a small cost validation study alongside our survey to gauge the accuracy and representativeness of our estimates.

The HRQoL for EV-A71 HFMD patients has only been reported in one study [8] which suggested that disability adjusted life years (DALY) incurred by an HFMD episode was 1.76/1000 for mild patients and 3.47/1000 for severe patients. Our HRQoL results are not directly comparable with theirs due to differences in methodology (e.g., in the design of survey instruments and utility measures) [26]. When compared with the health utility for children who had similar diseases, such as acute rotavirus gastroenteritis with same elicitation instrument, [27] mild and severe HFMD cases had higher utility score (0.83 and 0.61) than pediatric patients with acute rotavirus gastroenteritis (primary care cases: 0.69–0.78, severe cases 0.08–0.26). This suggested that HFMD had less impact on health than acute rotavirus gastroenteritis.

This study has a few major limitations. First, we did not retrieve individual medical records because China does not have a centralized electronic health records database. Instead, we asked interviewees to recall treatment costs, which were inevitably subject to recall bias. To investigate the likely magnitude of this bias, we compared the reported costs with medical records from a hospital in Xi’an. Our cost estimates were 22.7% (95% CI, 9.9%–35.6%) higher than that in the medical records. Second, because there are no instruments suitable for eliciting quality of life in young children, we used the proxy EQ-5D to assess the health utility and QALY loss.[28] We modified the adult version of the EQ-5D by fixing the responses to the “self-care” and “mobility” dimensions which were not applicable to very young children.[29] We gave these dimensions a rating of either “no problems” (which may underestimate HFMD impact) in the baseline analysis or “severe problems” in the sensitivity analyses. The sensitivity analysis results showed that the difference between the two extreme ratings had little influence on the HRQoL results for HFMD patients except for mild outpatients. Third, we used perfect health as the comparator when estimating QALY loss. In practice, the health status for the general population would not be perfect, hence we might have overestimated the QALY loss to some extent. Finally, our study only included cases recorded in the national HFMD surveillance system and hence ignored patients who did not seek medical treatment. Consequently, our cost and QALY loss estimates might be biased.

Despite these limitations, this study provides a comprehensive assessment of economic costs and HRQoL for HFMD patients in China. The first EV-A71 vaccines were licensed in December 2015 in China [6]. These EV-A71 vaccines are being considered for inclusion in the National Immunization Program in China due to heavy disease burden of HFMD[30]. Several studies have been conducted to support evidence-based decision making about the appropriate use of these vaccines in China. These include epidemiological studies of HFMD [4], transmission dynamic models to understand the impact of EV-A71 vaccination [31] and cost-effectiveness analysis of routine pediatric EV-A71 vaccination. [32] This study enriches the evidence base by providing nationally representative estimates of the economic costs and HRQoL of HFMD episodes of different severities which will serve as key inputs for future economic evaluation of HFMD vaccines in China.

## Supporting information

S1 File Calculation of QALY loss, weighted cost and QALY loss. (DOCX)

S2 File Medical accounts review. (DOCX)

S1 Table Questionnaire (brief version). (DOCX)

S2 Table Number of laboratory confirmed HFMD cases in seven districts for mild, severe and fatal groups in China, 2013, together with weights used to achieve geographical representativeness in economic outcomes. (DOCX)

S3 Table Economic costs per episode for HFMD patient in China, 2013 (US dollars). (DOCX)

S4 Table Economic burden for HFMD patients in the telephone survey. (DOCX)

S5 Table QALY loss for HFMD patients in the telephone survey. (DOCX)

S1 Fig Duration of illness for HFMD patients in each severity. (TIF)

S2 Fig Health utility elicited by EQ-5D for mild outpatient, mild inpatient and severe HFMD patients. (TIFF)

S3 Fig Health utility elicited by EQ-VAS for mild outpatient, mild inpatient and severe HFMD patients. (TIFF)
