# Supplementary material for: Economic costs and health-related quality of life for hand, foot and mouth disease (HFMD) patients in China
Source: PLoS One. 2017 Sep 21;12(9):e0184266. doi: 10.1371/journal.pone.0184266 (PMC5608208; doi:10.1371/journal.pone.0184266)
Supplement: S1 File — (DOCX) [file pone.0184266.s001.docx]

- **Calculation of QALY loss**

QALY loss for HFMD episode = (Health Utility _Full health_ -Health utility _HFMD episode_)ⅹduration of illness/365

- **Calculation of weighted cost**

$$The weighted total costs for mild outpatient HFMD patients=\sum(\frac{number of lab confirmed mild outpatient HFMD cases in one geographic region}{national number of lab confirmed mild outpatient HFMD cases}\times total cost for mild outpatient HFMD patients in one geographic region)$$

$$The weighted total costs for mild inpatient HFMD patients=\sum\frac{number of lab confirmed mild outpatient HFMD cases in one geographicregion}{national number of lab confirmed mild outpatient HFMD cases}\times total cost for mild inpatient HFMD patients in one geographic region$$

$$The weighted total costs for severe HFMD patients=\sum\frac{number of lab confirmed severe HFMD cases in one geographic region}{national number of lab confirmed severe HFMD cases}\times total cost for severe HFMD patients in one geographic region$$

$$The weighted total costs for fatal HFMD patients=\sum\frac{number of lab confirmed fatal HFMD cases in one geographic region}{national number of fatal HFMD cases}\times total cost for fatal HFMD patients in one geographic region$$

- **Calculation of weighted QALY loss**

$$The weighted QALY loss for mild outpatient HFMD patients=\sum\frac{number of lab confirmed mild outpatient HFMD cases in one geographic region}{national number of lab confirmed mild outpatient HFMD cases}\times QALY lossfor mild outpatient HFMD patients in one geographic \mathrm{region}$$

$$The weighted QALY loss for mild inpatient HFMD patients=\sum\frac{number of lab confirmed mild outpatient HFMD cases in one region}{national number of lab confirmed mild outpatient HFMD cases}\times QALY loss for mild inpatient HFMD patients in one geographic region$$

$$The weighted QALY loss for severe HFMD patients=\sum\frac{number of lab confirmed severe HFMD cases in one geographic region}{national number of lab confirmed severe HFMD cases}\times QALY loss for severe HFMD patients in\mathrm{one}geographic \mathrm{region}$$
