# Supplementary material for: Economic costs and health-related quality of life for hand, foot and mouth disease (HFMD) patients in China
Source: PLoS One. 2017 Sep 21;12(9):e0184266. doi: 10.1371/journal.pone.0184266 (PMC5608208; doi:10.1371/journal.pone.0184266)
Supplement: S2 File — (DOCX) [file pone.0184266.s002.docx]

- **Medical accounts review**

In our study cohort, a total of 115 HFMD patients reported that they received the inpatient treatment in Xi’an Pediatric Hospital. We randomly selected 70 out of 115 patients and ask a physician in HFMD special department in that hospital to search the hospitalization records manually. Totally, fifteen records were matched and included in our medical accounts review.

The demographic and costs for 15 HFMD patients in Xi’an pediatric hospital

| No. | Age | Gender | Urban/Rural | Record Cost | Report Cost | Difference In Cost |
| --- | --- | --- | --- | --- | --- | --- |
| 1 | 2.7 | male | urban | 6449 | 10001 | 36% |
| 2 | 4.9 | male | urban | 14250 | 16000 | 11% |
| 3 | 1.1 | female | urban | 9232 | 12000 | 23% |
| 4 | 2.4 | male | urban | 4788 | 5250 | 9% |
| 5 | 2.5 | male | urban | 11463 | 13000 | 12% |
| 6 | 4.1 | female | urban | 12441 | 13000 | 4% |
| 7 | 2.1 | male | urban | 13661 | 15000 | 9% |
| 8 | 1.6 | female | urban | 13136 | 13400 | 2% |
| 9 | 2.1 | male | rural | 2591 | 4500 | 42% |
| 10 | 2.7 | male | rural | 12264 | 12000 | -2% |
| 11 | 1.9 | male | urban | 13701 | 13900 | 1% |
| 12 | 2.6 | male | urban | 12998 | 15000 | 13% |
| 13 | 1.3 | female | urban | 12730 | 20001 | 36% |
| 14 | 1.2 | female | urban | 9926 | 12000 | 17% |
| 15 | 3.7 | female | urban | 12920 | 17800 | 27% |
