# Supplementary material for: Economic costs and health-related quality of life for hand, foot and mouth disease (HFMD) patients in China
Source: PLoS One. 2017 Sep 21;12(9):e0184266. doi: 10.1371/journal.pone.0184266 (PMC5608208; doi:10.1371/journal.pone.0184266)
Supplement: S1 Table — (DOCX) [file pone.0184266.s003.docx]

S1 Table Questionnaire (brief version)

| No. | Verifying below information (recorded in the HFMD surveillance network) before interviewing | |
| --- | --- | --- |
| V1 | Is the name of the patient xxx（the name of patients）？1) Yes 2）No | |
| V2 | Are you xxx（the name of patients or contacts persons of the patients）？1) Yes 2）No | |
| V3 | Address: | |
| V4 | Severity of patient: 1) mild; 2) severe; 3) fatal | |
| V5 | Age of patient: 1) six months- 1yr; 2) 1-2 yrs; 3) 2-3 yrs; 4) 3-4 yrs; 5) 4-5 yrs | |
| V6 | Interviewer’s name: xxx date of interview: yyyy/mm/dd length of interview: minutes | |
| No. | Questions and options | |
| 1 | How many times of HFMD have the patients had till now? | |
| 2 | Date of HFMD onset: yyyy/mm/dd | |
| 3 | Main diagnosis:  1)HFMD; 2) Hapangina; 3) Aseptic meningitis; 4) Encephalitis; 5) AFP; 6) respiratory infection; 7) Myocarditis; 8) Pulmonary hemorrhage; 9) Pulmonary edema; 10) Cardiopulmonary failure; 11) others | |
| 4 | Did the child have other underlying disease besides the disease mentioned in question 3? | |
| 5 | The duration of the HFMD: | |
| 6 | How many times of outpatient treatments did the patient receive for HFMD? | |
| 7 | Here is the question example for one time treatment (if beyond 1 time, please answer question 7 for each time) | |
|  | 7.1 Name of the hospital where you seek medical care |  |
|  | 7.2 How much do you spend on medication？ | Yuan  If the interviewee cannot recall the exact cost, please ask him/her to select an option below:  1) <100; 2) 100-299; 3) 300-499; 4) 500-799; 5) 800-999; 6) 1000-1999; 7) 2000-2999; 8) ≥3000 |
|  | 7.3 How much do you (including accompanying persons) spend on transportation？ | Yuan  If the interviewee cannot recall the exact cost, please ask him/her to select an option below:  1) <10; 2) 10-29; 3) 30-49; 4) 50-99; 5)100-199; 6) ≥200; 7) If you drove to hospitals and cannot estimate the cost, please tell me the distance between your home and the hospital: kilometers，and the type of vehicles：a.car, b.motorbike, c.bike, d.others |
|  | 7.4 How many persons accompany you to hospitals（If zero, please skip to Question 8） | persons |
|  | 7.5 How many days did a person accompany you on average? | days |
| 8 | Were you hospitalized during that episode？1）Yes 2）No （if no，skip to Question 9） | |
| 9 | How many times were you hospitalized during that episode? | |
| 10 | Here is the question example for one time treatment (if beyond 1 time, please answer question 10 for each time) | |
|  | 10.1 Name of the hospital where you seek medical care |  |
|  | 10.2 How many days were you hospitalized | days |
|  | 10.3 How much do you spend on medication？ | Yuan  If the interviewee cannot recall the exact cost, please ask him/her to select an option below:  1) <1000; 2) 1000-1999; 3) 2000-2999; 4) 3000-3999; 5) 4000-4999; 6) 5000-9999; 7) 10000-19999; 8) 20000-29999; 9) ≥30000 |
|  | 10.4 How many persons accompany you at hospitals（If zero, please skip to Question 10.8） | persons |
|  | 10.5 How many days did a person accompany you on average? | days |
|  | 10.6 How much did an accompanying person spend on accommodation each day on average? | Yuan/Days  If the interviewee cannot recall the exact cost, please ask him/her to select an option below:  1) <100; 2) 100-199; 3) 200-299; 4)300-399; 5)400-499; 6) ≥500 |
|  | 10.7 How much did an accompanying person spend on meals each day on average? | Yuan/Days  If the interviewee cannot recall the exact cost, please ask him/her to select an option below:  1) <20; 2) 20-49; 3) 50-99; 4)100-199; 5)200-299; 6) ≥300 |
|  | 10.8 How much did you spend on your own meals each day on average? | Yuan/Days  If the interviewee cannot recall the exact cost, please ask him/her to  select an option below:  1) <20; 2) 20-49; 3) 50-99; 4)100-199; 5)200-299; 6) ≥300 |
|  | 10.9 Did you hire a caregiver？If yes, how much did you spend on it? If no, please fill in zero. | Yuan  If the interviewee cannot recall the exact cost, please ask him/her to select an option below:  1) <200 ; 2)200-499 ; 3) 500-999; 4) 1000-1499 ; 5) 1500-1999; 6) ≥2000 |
|  | 10.10 How much do you (including accompanying persons) spend on transportation？ | Yuan  If the interviewee cannot recall the exact cost, please ask him/her to select an option below:  1) <100 ; 2) 100-199 ; 3) 200-299; 4) 300-499 ; 5) 500-999 ; 6) ≥1000; 7) If you drove to hospitals and cannot estimate the cost, please tell me the distance between your home and the hospital: kilometers, and the type of vehicles：a.car, b.motorbike, c.bike, d.others |
| 9 | How much did you spend on self-medications for the episode of HFMD? | Yuan  If the interviewee cannot recall the exact cost, please ask him/her to select an option below:  1) <30 ; 2) 30-49; 3) 50-99; 4)100-199; 5) 200-499; 6)500-999; 7) ≥1000 |
| 10 | How much did you spend on nourishment for the episode of HFMD? | Yuan  If the interviewee cannot recall the exact cost, please ask him/her to select an option below:  1) <30 ; 2) 30-49; 3) 50-99; 4)100-199; 5) 200-499; 6)500-999; 7) ≥1000 |
| No. | Questions and options | |
| 1 | Mobility   1. No problem 2. Some problem 3. Extreme problem | |
| 2 | Usual activity   1. No problem 2. Some problem 3. Extreme problem | |
| 3 | Pain/discomfort   1. No problem 2. Some problem 3. Extreme problem | |
| 4 | Depression/anxiety   1. No problem 2. Some problem 3. Extreme problem | |
| 5 | Self-care (neglected)   1. No problem 2. Some problem 3. Extreme problem | |
| 6 | Please imagine that is a scale for evaluating the health state for child, 100 means full health, 0 means the worst. Can you give the score for child when he/she in the disease state of HFMD? | |
