# Supplementary material for: Economic costs and health-related quality of life for hand, foot and mouth disease (HFMD) patients in China
Source: PLoS One. 2017 Sep 21;12(9):e0184266. doi: 10.1371/journal.pone.0184266 (PMC5608208; doi:10.1371/journal.pone.0184266)
Supplement: S2 Table — (DOCX) [file pone.0184266.s004.docx]

Number of laboratory confirmed HFMD cases in seven districts for mild, severe and fatal groups in China, 2013, together with weights used to achieve geographical representativeness in economic outcomes.

| No. | District | Mild | | severe | | Fatal | |
| --- | --- | --- | --- | --- | --- | --- | --- |
|  |  | Number | Weight | Number | Weight | Number | Weight |
| 1 | Number of lab confirmed HFMD cases in Northeast of China | 4759 | 0.06 | 174 | 0.03 | 6 | 0.04 |
| 2 | Number of lab confirmed HFMD cases in North China | 8635 | 0.10 | 243 | 0.05 | 20 | 0.12 |
| 3 | Number of lab confirmed HFMD cases in East China | 22445 | 0.27 | 1392 | 0.28 | 29 | 0.18 |
| 4 | Number of lab confirmed HFMD cases in South China | 11389 | 0.14 | 489 | 0.10 | 23 | 0.14 |
| 5 | Number of lab confirmed HFMD cases in Central China | 18478 | 0.22 | 775 | 0.15 | 28 | 0.17 |
| 6 | Number of lab confirmed HFMD cases in Northwest of China | 4924 | 0.06 | 424 | 0.08 | 4 | 0.02 |
| 7 | N Number of lab confirmed HFMD cases | 11659 | 0.14 | 1555 | 0.31 | 52 | 0.32 |
|  | National number of lab confirmed HFMD cases | 82289 | 1.00 | 5052 | 1.00 | 162 | 1.00 |

Source: National enhanced HFMD surveillance database
