# Supplementary material for: Economic costs and health-related quality of life for hand, foot and mouth disease (HFMD) patients in China
Source: PLoS One. 2017 Sep 21;12(9):e0184266. doi: 10.1371/journal.pone.0184266 (PMC5608208; doi:10.1371/journal.pone.0184266)
Supplement: S3 Table — (DOCX) [file pone.0184266.s005.docx]

S3 Table. Economic costs per episode for HFMD patient in China, 2013 (US dollars)

|  | Mild outpatient  N=1136 | | Mild inpatient  N=1124 | | Severe  N=1170 | | Fatal  N=61 | |
| --- | --- | --- | --- | --- | --- | --- | --- | --- |
|  | Median  (p25,p75) | Mean  (95%CI) | Median  ((p25,p75) | Mean  (95%CI) | Median  ((p25,p75) | Mean  (95%CI) | Median  ((p25,p75) | Mean  (95%CI) |
| Direct medical cost | 60  (24,113) | 88  (82,94) | 403  (242,807) | 759  (695,823) | 2091  (1496,2992) | 2513  (2379,2648) | 1330  (807,2685) | 2148  (1531,2766) |
| Direct non-medical cost | 32  (8,65) | 49  (45,54) | 187  (109,303) | 248  (235,261) | 308  (182,524) | 420  (399,441) | 245  (110,598) | 462  (321,602) |
| Indirect cost | 10  (0,69) | 64  (54,73) | 26  (0,83) | 65  (58,72) | 64  (0,157) | 117  (107,128) | 39  (0,143) | 209  (52,367) |
| Total cost for all HFMD cases | 135  (63,259) | 201  (187,215) | 678  (450,1161) | 1072  (999,1144) | 2497  (1836,3637) | 3051  (2905,3197) | 1564  (1113,3986) | 2819  (2068,3571) |
| Weighted total cost | 168  (106,217) | 196  (75,318) | 835  (387,1763) | 990  (431,1549) | 1830  (965,5996) | 3084  (813,5354) | 2680  (1068,3763) | 2348  (1006,3689) |
