# Supplementary material for: Economic costs and health-related quality of life for hand, foot and mouth disease (HFMD) patients in China
Source: PLoS One. 2017 Sep 21;12(9):e0184266. doi: 10.1371/journal.pone.0184266 (PMC5608208; doi:10.1371/journal.pone.0184266)
Supplement: S4 Table — (DOCX) [file pone.0184266.s006.docx]

S4 Table. Economic burden and influential factors for HFMD patients

|  | Mild outpatient | | |  | Mild inpatient | | |  | Severe | | |  | Fatal | | |
| --- | --- | --- | --- | --- | --- | --- | --- | --- | --- | --- | --- | --- | --- | --- | --- |
|  | Median  (p25,p75) | Mean | MLR with Bootstrapping  Coefficient (95% CI) |  |  | Mean | MLR with Bootstrapping  Coefficient (95% CI) |  |  | Mean | MLR with Bootstrapping  Coefficient (95% CI) |  | Median  (p25,p75) | Mean | MLR with Bootstrapping  Coefficient (95% CI) |
| Gender |  |  |  |  |  |  |  |  |  |  |  |  |  |  |  |
| Male | 136  (65,253) | 204  186,223) | Reference |  | 680  (455,1152 | 1065  (976,1155) | Reference |  | 2489  (1824,3645) | 3037  (2849,3224) | Reference |  | 1928  (1113, 5340) | 3362  (2179,4546) | Reference |
| female | 132  (56,273) | 195  (173,217) | -14  (-40,13) |  | 672  (438,1201) | 1084  (960,1207) | -20  (-149,108) |  | 2524  (1888,3601) | 3079  (2851,3308) | 32  (-260,324) |  | 1380  (1088, 2739) | 2089  (1328,2849) | -467  (-1852,917) |
| Age group |  |  |  |  |  |  |  |  |  |  |  |  |  |  |  |
| ≤3 years | 129(60,260) | 200  (183,216) | Reference |  | 738(466,1252) | 1149  (1054,1245 | Reference |  | 2545  (1831,3637) | 3103  (2919,3287) | Reference |  | 1805  (1136,5186) | 3169  (2161,4177) | Reference |
| >3 years | 145(66,256) | 203  (177,229) | 2  (-26,31) |  | 613(409,993) | 904  (806,1002) | -189  (-313,-65)** |  | 2470  (1867,3623) | 2898  (2701,3095) | -252  (-503,-1)* |  | 1332  (1088,2560) | 1915  (1278,2552) | -2477  (-4324,-630)** |
| Geographic regions |  |  |  |  |  |  |  |  |  |  |  |  |  |  |  |
| Northeast | 161(79,323) | 252  (210,295) | Reference |  | 685  (485,1088) | 922  (810,1034) | Reference |  | 2062  (1381, 3651) | 2769  (2114,3424) | Reference |  | 4069  (2770,5198) | 3924  (2802,5047) | Reference |
| North west | 145(65,232) | 189  (152,227) | -52  (-106,1) |  | 488  (346,845) | 849  (642,1057) | 27  (-215,270) |  | 2641  (2143,3283) | 2820  (2590,3051) | -12  (-619,595) |  | 506  (502,1284)) | 764 (-354,1883) | -2302  (-5521,917) |
| North | 118(55,245) | 191  (158,225) | -49  (-102,4) |  | 1219  (717,2174) | 1733  (1548,1918) | 741  (540,942)*** |  | 2316  (1612, 2301) | 2757  (2406,3108) | 50  (-570,669) |  | 2106  (1429,2782) | 2106  (-6491,10703) | -2190  (-4835,454) |
| Central | 100(44,206) | 141  (120,163) | -99  (-143,-54) *** |  | 623  (436,1111) | 1149  (847,1450) | 260  (-30,549) |  | 3052  (2062,4460) | 3877  (3346,4407) | 662  (-75,1398) |  | 1430  (1150,2739) | 2498  (-231,5227) | 1098  (-1833,4029) |
| Southwest | 92(42,254) | 171  (140,202) | -59  (-109,-10)* |  | 493  (298, 776) | 617  (523,710) | -199  (-354,-43)* |  | 2427  (1823, 3593) | 3025  (2769,3281) | 155  (-452,761) |  | 1332  (1116,1932) | 1644  (1139,2150) | -30  (-2047,1986) |
| East | 173(98,359) | 251  (212,290) | 12  (-43,68) |  | 691  (514,1085) | 933  (811,1055) | 143  (-21,306) |  | 2860  (1937,3856) | 3059  (2812,3305) | 138  (-474,751) |  | 1032  (689,5340) | 3496 (-285,7278) | 3154  (-1151,7459) |
| South | 132(76,257) | 201  (156,247) | -32  (-93,29) |  | 585  (421,891) | 852  (667,1038) | 28  (-183,238) |  | 2163  (1496, 2932) | 2614  (2271,2957) | -153  (-814,509) |  | 1682  (1113,4398) | 3042  (1284,4800) | 1049  (-1558,3656) |
| Etiology |  |  |  |  |  |  |  |  |  |  |  |  |  |  |  |
| EV-A71^$^ | 125  (52,242) | 192  (171,212) | Reference |  | 890  (486,1792) | 1420  (1274,1567) | Reference |  | 2666  (1956,3813) | 3233  (2802) | Reference |  | 1550  (1113,3986) | 2854  (2023,3684) | Reference |
| CV-A16 | 125  (63,215) | 198  (157,238) | 19  (-9,47) |  | 590  (422,884) | 742 (665,819) | -462  (-611,-314)*** |  | 2389  (1439,2946) | 2781  (2227,3336) | -354  (-621,-87)** |  | 476  (476,476) | 476  (-) | -1896  (-4721,929) |
| OEV | 150  (70,283) | 211  (190,232) | -1  (-44,41) |  | 613  (426,901) | 832 (762,901) | -512  (-661,-363)*** |  | 2290  (1612,3111) | 2642 (2445,2840) | -338  (-773,98) |  | 2351  (1167,5340) | 2903  (792,5013) | -3285  (-6342,-227)* |
| Duration of illness/days |  |  |  |  |  |  |  |  |  |  |  |  |  |  |  |
| ≤5^$^ | 101  (49,185) | 145  (122,167) | Reference |  | 462  (273,767) | 804  (367,1242) | Reference |  | 1499  (975, 1904) | 1772  (1280,2264) | Reference |  | 1178  (765, 1727) | 1556  (958,2153) | Reference |
| 6-10 | 128  (65,246) | 192  (176,208) | 48  (21,74) *** |  | 605  (420,925) | 813  (756,870) | -112  (-535,311) |  | 1981  (1318, 2545) | 2171  (1997,2345) | 394  (-133,921) |  | 1932  (1150, 4398) | 3470  (1414,5526) | 3923  (708,7138)* |
| 11-15 | 207  (97,365) | 246  (214,279) | 104  (64,144) *** |  | 975  (589,1664) | 1324  (1176,1472) | 333  (-114,780) |  | 2689  (2042,3603) | 2982  (2818,3145) | 1134  (617,1651)*** |  | 1783  (1167, 2770) | 2084  (1106,3062) | 3624  (67,7180)* |
| 16-20 | 250  (113,415) | 340  (209,471) | 188  (59,317)** |  | 1089  (590,2352) | 1884  (1466,2301) | 859  (292,1427)** |  | 3434  (2495, 4735) | 3917  (3541,4292) | 1929  (1285,2573) *** |  | 6442  (4402, 9411) | 6890  (4085,9696) | 6837  (3891,9784)*** |
| ≥21 | 323  (152,567) | 478  (212,743) | 329  (76,582)* |  | 2376  (1053, 3774) | 2823  (1996,3649) | 1723  (860,2586)*** |  | 4443  (3052, 5974) | 5151  (4359,5942) | 3215  (2355,4075) *** |  | 3581  (2020,4717) | 3369  (404,6333) | 4716  (631,8801)* |

*P<0.05, **P<0.01, ***P<0.001

MLR: Multiple linear regression. EV-A71: enterovirus A 71, CV-A16: coxsackievirus A 16. OEV: other enterovirus.
