# Supplementary material for: Economic costs and health-related quality of life for hand, foot and mouth disease (HFMD) patients in China
Source: PLoS One. 2017 Sep 21;12(9):e0184266. doi: 10.1371/journal.pone.0184266 (PMC5608208; doi:10.1371/journal.pone.0184266)
Supplement: S5 Table — (DOCX) [file pone.0184266.s007.docx]

QALY loss for HFMD patients in the telephone survey

|  | Mild outpatient | | | Mild inpatient | | | Severe | | |
| --- | --- | --- | --- | --- | --- | --- | --- | --- | --- |
|  | Median(p25,p75) | Mean(95%CI) | P-value | Median(p25,p75) | Mean(SD) | P-value | Median(p25,p75) | Mean(SD) | P-value |
| Gender |  |  | 0.049 |  |  | 0.572 |  |  | 0.3158 |
| Male | 2.5(0, 4.8) | 3.4(3.1, 3.9) |  | 4.8(2.5, 8.8) | 6.8(6.2, 7.4) |  | 9.6(4.8, 17.8) | 13.4(12.4, 14.5) |  |
| female | 3.0(1.4, 4.9) | 3.9(3.5, 4,4) |  | 4.8(2.7, 8.4) | 7.1(6.3, 7.9) |  | 10.1(5.6,20.0) | 14.3(13.0, 15.6) |  |
| Age group |  |  | 0.005 |  |  | 0.191 |  |  | 0.506 |
| ≤3 years | 3.0(1.4, 5.0) | 3.9(3.5, 4.2) |  | 4.8(2.7, 8.9) | 7.1(6.6, 7.7) |  | 9.6(5.1, 18.5) | 13.6(12.6, 14.5) |  |
| >3 years | 2.4(0, 4.2) | 3.1(2.8, 3.5) |  | 4.2(2.4, 8.0) | 6.5(5.5, 7.4) |  | 10.4(5.4, 18.9) | 14.2(12.6, 15.8) |  |
| Districts |  |  | 0.026 |  |  | <0.001 |  |  | 0.096 |
| Northeast | 3.0(0, 5.6) | 4.4(3.5,5,3) |  | 4.3(2.8, 8.2) | 6.2(5.4, 7.0) |  | 10.7(4.4, 18.9) | 17.8(6.5, 29.0) |  |
| North west | 3.6(0.5, 5.6) | 4.0(3.4, 4.7) |  | 5.4(2.6, 8.3) | 7.2(5.4, 9,0) |  | 8.7(4.2, 18.6) | 12.5(11.0, 14.0) |  |
| North China | 2.4(0, 4.2) | 3.0(2.5, 3.5) |  | 5.7(3.0, 10.7) | 8.9(7.7, 10.1) |  | 9.5(5.0, 15.6) | 12.3(10.3, 14.3) |  |
| Central China | 2.5(0, 4.2) | 3.1(2.6, 3.6) |  | 4.2(2.4, 7.5) | 6.9(5.2, 8.6) |  | 11.9(5.9, 21.8) | 15.5(13.8, 17.2) |  |
| Southwest | 2.4(1.2, 4.2) | 3.5(2.8, 4.1) |  | 4.8(2.5, 8.4) | 6.1(5.2, 7.0) |  | 10.2(5.0, 19.0) | 14.2(12.4, 15.9) |  |
| East China | 3.6(1.4, 5.1) | 3.7(3.1, 4.3) |  | 4.2(2.4, 7.3) | 5.4(4.5, 6.3) |  | 9.6(5.6, 15.0) | 12.7(11.0, 14.3) |  |
| South China | 2.4(1.0, 4.2) | 3.4(2.7, 4.1) |  | 4.2(2.4, 8.0) | 6.6(5.4, 7.8) |  | 8.9(4.2, 18.0) | 13.3(11.2, 15.3) |  |
| Lab test |  |  | 0.6827 |  |  | <0.001 |  |  | <0.001 |
| EV-A71 | 2.5(0, 4.8) | 3.6(3.2, 4.0) |  | 5.6(2.7, 10.2) | 8.2(7.4, 9.1) |  | 10.7(5.6, 19.4) | 14.9(13.9, 16.0) |  |
| CV-A16 | 3.0(1.7, 5.0) | 3.9(3.2, 4.5) |  | 4.2(2.5, 8.3) | 6.2(5.3, 7.1) |  | 8.1(3.9, 15.9) | 11.8(8.1, 15.4) |  |
| OEV | 2.5(0, 4.8) | 3.6(3.2, 3.9) |  | 4.2(2.4, 7.3) | 5.8(5.2, 6.4) |  | 7.5(4.2, 14.2) | 11.0(10.3) |  |
| Duration of illness/days |  |  | <0.001 |  |  | <0.001 |  |  | <0.001 |
| ≤5 | 1.1(0, 2.4) | 1.2(1.0, 1.4) |  | 1.8(1.2, 2.4) | 1.8(1.5,2.2) |  | 2.4(1.5, 4.4) | 2.8(2.0, 3.7) |  |
| 6-10 | 3.0(1.7, 4.2) | 3.1(2.9, 3.3) |  | 4.2(2.4, 5.6) | 4.3(4.1, 4.6) |  | 5.6(3.6, 9.6) | 6.8(6.4, 7.2) |  |
| 11-15 | 5.9(3.6, 8.0) | 6.1(5.4, 6.8) |  | 8.0(5.9, 12.9) | 9.8(8.9, 10.7) |  | 10.7(5.9, 18.1) | 12.1(11.3, 12.8) |  |
| 16-20 | 8.9(5.4, 12.0) | 9.6(7.5,11.6) |  | 10.9(8.9, 19.9) | 14.7(12.8, 16.7) |  | 18.8(12.0, 27.9) | 19.9(18.5, 21.3) |  |
| ≥21 | 19.6(7.2, 19.6) | 15.5(9.4,21.6) |  | 21.4(14.9, 35.5) | 27.0(19.4, 34.6) |  | 31.6(17.5, 45.0) | 34.7(29.9, 39.6) |  |

EV-A71: enterovirus A 71, CV-A16: coxsackievirus A 16. OEV: other enteroviru
