# Supplementary figures and images for: Economic costs and health-related quality of life for hand, foot and mouth disease (HFMD) patients in China
Source: PLoS One. 2017 Sep 21;12(9):e0184266. doi: 10.1371/journal.pone.0184266 (PMC5608208; doi:10.1371/journal.pone.0184266)

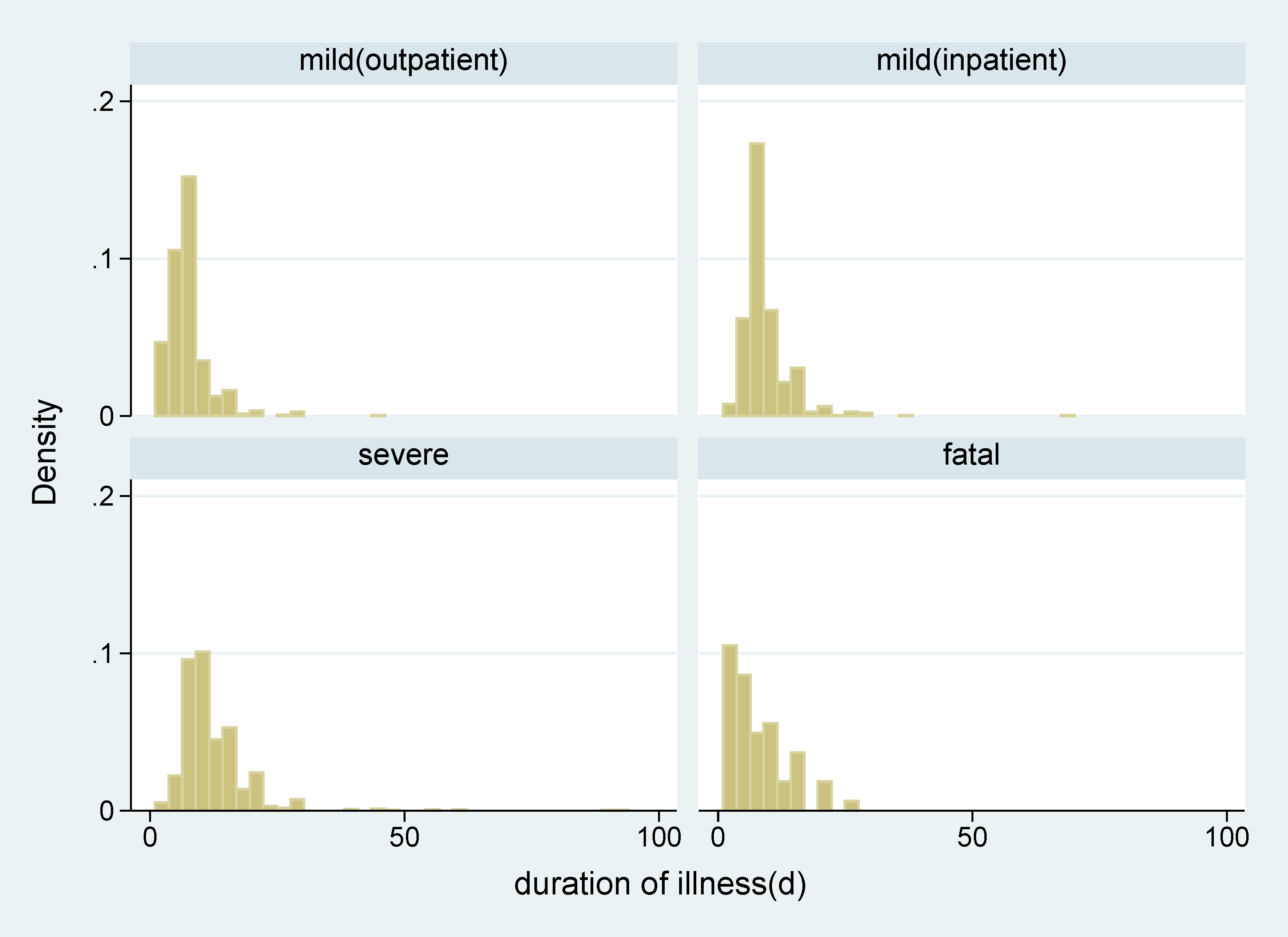

Supplement: S1 Fig — (TIF) [file pone.0184266.s008.tif]

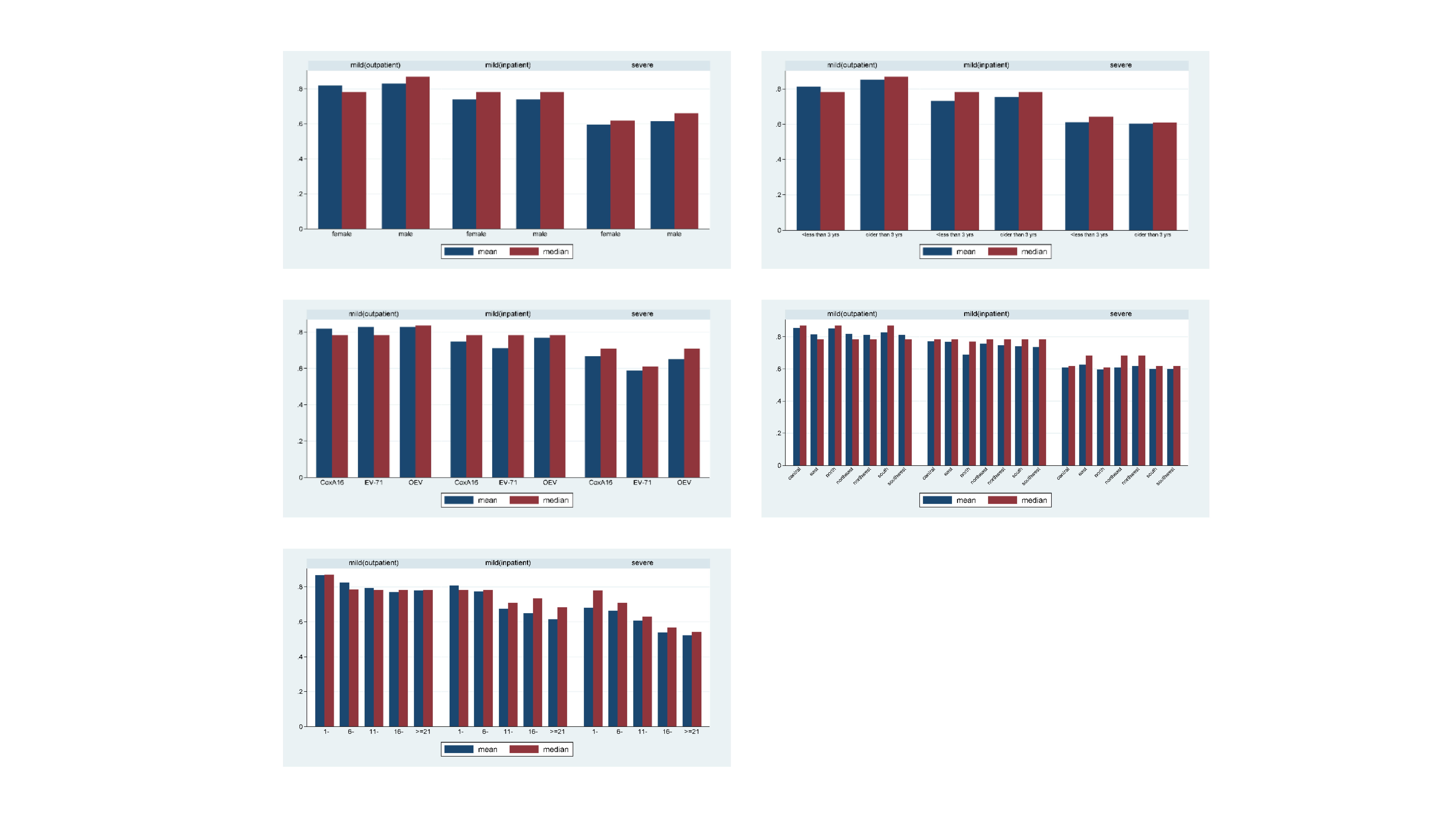

Supplement: S2 Fig — (TIFF) [file pone.0184266.s009.tiff]

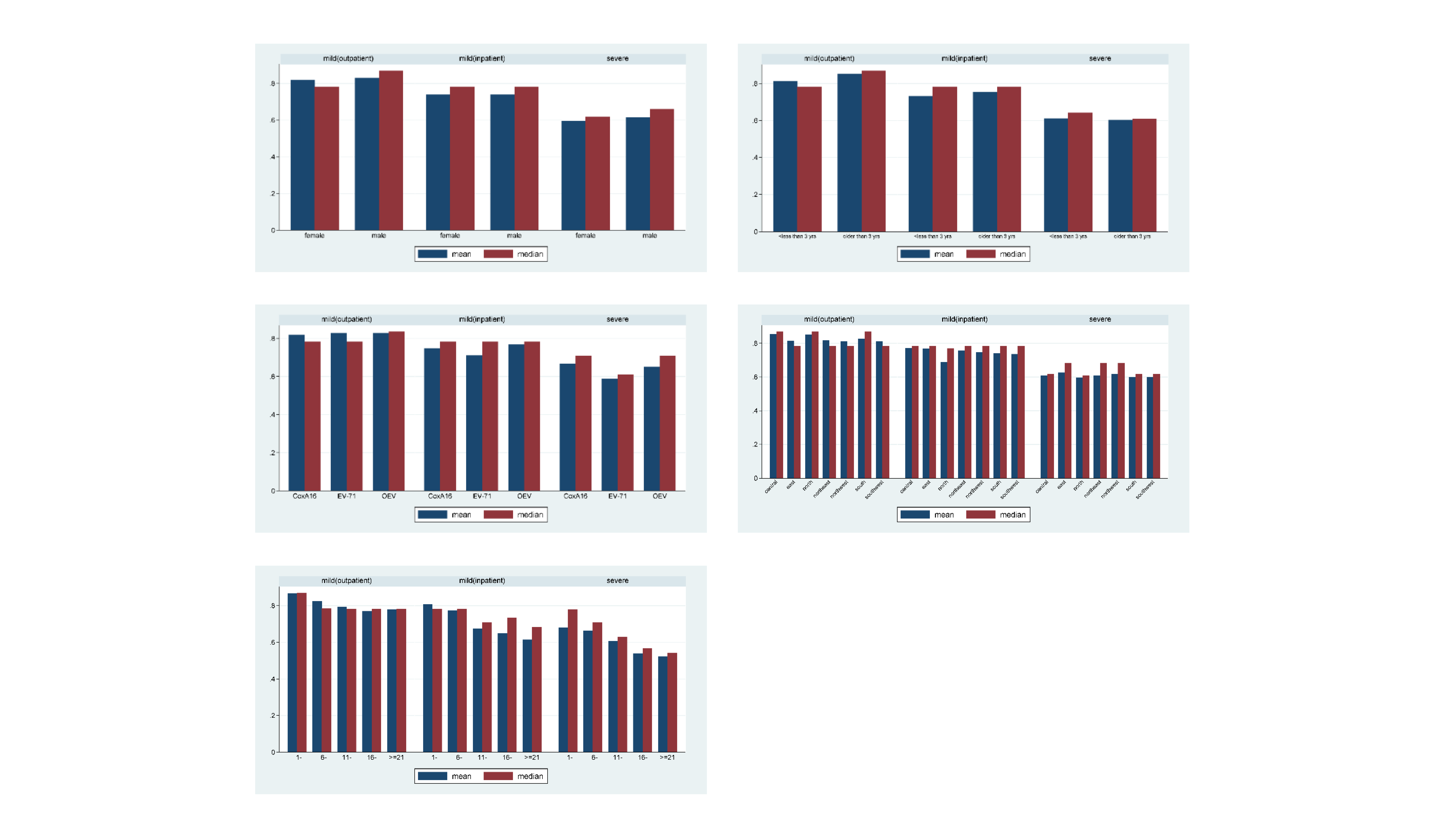

Supplement: S3 Fig — (TIFF) [file pone.0184266.s010.tiff]
